# Accessible and Inclusive Cyber Security: A Nuanced and Complex Challenge

**DOI:** 10.1007/s42979-022-01239-1

**Published:** 2022-06-22

**Authors:** Karen Renaud, Lizzie Coles-Kemp

**Affiliations:** 1 University of Strathclyde, Glasgow, UK Rhodes University, Grahamstown, South Africa University of South Africa, Pretoria, South Africa Abertay University, Dundee, UK; 2 Royal Holloway University of London, Egham, UK

**Keywords:** Accessibility, Cyber security, Vulnerability

## Abstract

It has been argued that human-centred security design needs to accommodate the considerations of three dimensions: (1) security, (2) usability and (3) accessibility. The latter has not yet received much attention. Now that governments and health services are increasingly requiring their citizens/patients to use online services, the need for accessible security and privacy has become far more pressing. The reality is that, for many, security measures are often exasperatingly inaccessible. Regardless of the outcome of the debate about the social acceptability of compelling people to access public services online, we still need to design accessibility into these systems, or risk excluding and marginalising swathes of the population who cannot use these systems in the same way as abled users. These users are particularly vulnerable to attack and online deception not only because security and privacy controls are inaccessible but also because they often struggle with depleted resources and capabilities together with less social, economic and political resilience. This conceptual paper contemplates the accessible dimension of human-centred security and its impact on the inclusivity of security technologies. We scope the range of vulnerabilities that can result from a lack of accessibility in security solutions and contemplate the nuances and complex challenges inherent in making security accessible. We conclude by suggesting a number of avenues for future work in this space.

## Introduction

In a recent paper, Renaud [73] argued that accessibility ought to be considered an essential third dimension of the cyber security design domain, in addition to technical security and usability design considerations. It is essential that security be assured and not compromised by usability and accessibility enhancement efforts [41]. While security and usability are well established considerations in technology design, accessibility has not yet received as much attention. We are not the first to call for attention to be paid to accessibility. For example, Wang  [113], in 2017, raised the need for inclusivity in security and privacy solutions. In this paper, we extend the argument for accessibly secure technologies and services. Accessible systems are inclusive: not only do inaccessible systems exclude those with disabilities, but such systems are also designed in a way that does not acknowledge the social and economic precarities that often shape living with disabilities [83]. Understanding how these social, economic and even political insecurities intersect with digital insecurity is important if the strength of controls and the nature of digital vulnerabilities are to be truly understood and accommodated during the design process.

There is a need for the issue of accessibility in security and privacy technology design to gain greater prominence, given that many governments and health services are planning a future where primary care is delivered via a blended service. Health care is something that everyone needs sooner or later so people will have no choice but to engage with these online services. These services aim to utilise digital and tele-health services to deliver health care [85, 96, 102]. Rosner et al. [80] make the point that “*Nothing about us without us*” (p. 5) and point to the urgency of ensuring inclusivity in our interface designs. Yet, there does not seem to be any debate about which technological innovations are socially acceptable and fair to impose on citizens [57], so the march to force people into using online services proceeds apace.

People have no choice when they are dependent on these digital services for essential, everyday access—such as financial, housing, welfare and educational services. Moreover, whereas vulnerable people are often well supported in the physical world to assure accessibility and inclusivity, the online world often does not have the same structures in place.

Cyber security is a case in point, where it is often assumed that users are fully abled (e.g. can see the CAPTCHA), cognitively unimpaired (e.g. can create and retain passwords), have the necessary resources (e.g. time, appropriate technology and internet access in a distraction-free environment), and have the required dexterity to interact with the security system (e.g. can use the mouse and keyboard with ease). Other aspects such as low self-efficacy perceptions or low self confidence can also constitute barriers to adoption of data and technology protection practices. The consequence is exclusion from essential online services. This cannot continue.

This paper is essentially conceptual, highlighting the nuances and challenges of giving accessibility its rightful place in the cyber security domain. Our work contemplates the nuances and complex challenges designers face in improving the accessibility and inclusivity of cyber security solutions.

Carter and Markel [16] argue that the most promising route to full accessibility lies in collaboration between vendors, advocacy groups, and the government. We write this paper in the hope of triggering exactly such a discourse involving cyber security professionals, human-centred security academics, disability charities and other stakeholders. The idea is to highlight the emerging and inescapable need to consider accessibility as being as important as security and usability considerations in the cyber security field.

We first talk about the inclusive security movement more broadly in Sect. 2 and then set out the concept of accessibility in Sect. 3. Section 4 will consider accessibility challenges experienced by people following government-provided advice for data and technology protection practices (often termed cyber hygiene advice). Section 5 then suggests a path forward in terms of meeting the challenges of this domain. With Sect. 6, we conclude the paper.

## Inclusive Security

The move towards accessible security and privacy technology design needs to be understood within the wider context of inclusive security. As a concept, inclusive security is seemingly at odds with the more traditional framing of security which focuses on prevention and exclusion [25]. However, in the digital context, security has increasingly had an inclusive dimension and *inclusive security and privacy* has become an area of study in its own right [113]. From a security philosophy point of view, an inclusive security outlook in technology design focuses on security as enablement, the ability to live free from fear of security threats in their broadest sense. Such a way of viewing security is often termed “positive security” [25, 79]. Positive security contrasts with the more traditional outlook of security as protection from harms and threats, termed “negative security” [25, 79].

Accessible security and privacy technologies not only enable a broader cross-section society to access digital services free from the fear of digital threats and attacks, but also enable people to access essential digital services. Access to such services enable people to live free from fear of living without access to the resources needed to build a secure life. As societies become ‘digital by default’ or ‘digital first’, it has become a wider societal risk if individuals are unable to access statutory services. With this move, there is an increasing tendency for many governments to deliver essential, everyday services such as welfare, healthcare, finance, education and transport services using digital means. During the COVID-19 pandemic, digital delivery has often replaced rather than augmented face-to-face delivery. Lack of access to essential services can undermine societal cohesion and therefore also societal security. Inclusive security ensures that access to such services are accessible at the point of need, and provide digital protections to the individual using the service regardless of the individual’s capabilities, abilities and resources. Equally, it is also important that when using the service both the technology design and the underlying service logic do not exacerbate the insecurities and vulnerabilities of the individual using the service. Accessibility issues are a major concern for those that are digitally excluded. In the following subsection we outline what digital exclusion means and the implications for the use of security and privacy technologies.

### Digital Exclusion

Those who are digitally excluded often find security and privacy technologies inaccessible. Digital exclusion is multifaceted and is typically considered from three perspectives [105]:

1. Physical access to digital devices.
2. Skills to navigate the digital world.
3. Inequalities of access.

*Digital Exclusion in Three Perspectives:*

Exclusion is typically characterised as a form of digital divide and this divide might be defined as “*the gap between people who do and do not have access to forms of information and communication technology*”  [106]. Access to technology is contingent on physical access to security technology and the availability of the underpinning technical and data infrastructure. However there are additional contingencies to consider: access is not equitable if people cannot afford the technology, or if access to the service is designed in such a way that there is hostility or suspicion to particular marginalised groups of users, or they do not have the skills, capabilities and resources needed to access those services [20] (see Sects. 3.1.1, 3.1.2, 3.1.3). These contingencies are sometimes referred to as being beyond access and as the second-level divide [106]. These perspectives reveal that there are a number of ways in which security and privacy technologies might be inaccessible to those experiencing digital exclusion. The digital exclusion framework shows that digital exclusion can take many forms and the different dimensions of digital exclusion need to be considered when designing, evaluating and deploying security and privacy technologies.

Digital exclusion is often (but not always) experienced by marginalised and underserved groups, exacerbating their vulnerabilities and precarity. In the following subsection we consider digital security in the context of marginalised and underserved groups.

### Digital Security and Marginalised and Underserved Groups

Inclusive security design requires an approach that is attentive to the security needs of different communities and to the challenges that different communities have in using security technologies. Briggs and Thomas conducted 12 workshops with 91 participants from 6 marginalised groups to evaluate the challenges and barriers to identity management [12]. In this process, Briggs and Thomas aimed to identify the digital identity requirements responding to as a diverse a set of needs as possible. They identified a series of design features and affordances that are likely to result in digital identity being accepted as a technology by many groups across society. One of the ways in which security design can be made more inclusive is by addressing a broader range of vulnerabilities [91] and by identifying and understanding threats from different perspectives [43]. Studies in intimate partner violence [30, 53, 69] and refugee studies [1, 21, 98] have particularly driven this more broader understanding of security threats and the digital protection needs of groups that face heightened vulnerabilities, often for a prolonged period of time. The issues experienced by refugees and those experiencing intimate partner violence are also experienced by other marginalised and underserved groups. Surveillance, lack of access to essential services, privacy violations, misuse of technology to gain advantage and exert power over another are all issues that are experienced by many marginalised and underserved groups. Threat modelling has been identified as an activity that can bring security technologists and marginalised and underserved groups into conversation with the possibility of more inclusive security technologies emerging as a result. As media studies scholar, Kazansky highlights understanding different perspectives on threats also requires broadening our understanding of what constitutes security practices and recognition that some of the security solutions developed by technologists can exacerbate the vulnerabilities of marginalised groups [2, 43]. Threat modelling offers a means of anchoring abstract security problems and threats to a particular context and a key means by which the meanings of security technology for a particular community can be made clear [43, 92]. A critical examination of the practice of threat modelling has resulted in inclusive and participatory approaches to the practice to ensure that the experiences of marginalised and underserved communities are centred in the security analysis of different digital contexts [91].

Such an inclusive approach has the potential to lead to a wider appreciation of how different communities can be made vulnerable through technology practices and design. For example, HCI scholar Strohmeyer undertook studies with sex workers to better understand the information sharing and protection practices that this community undertakes to protect themselves in their work life [97]. In another example, use of augmentative alternative communication (AAC) technologies [10] has been further explored to better understand security and privacy issues and responses by AAC users and their support network [71]. Such understanding leads to the possibility of designing technologies that better support these practices or that learn from these practices to re-design the ways in which security and privacy technologies are incorporated into communications technologies.

### Understanding the Intersections

An inclusive security approach also requires that security technologists understand the complexities that often surround digital access in marginalised and underserved groups and how this impacts the use of security and privacy technologies. Digital vulnerabilities and how people respond to them do not exist in a vacuum. The relative strength of controls and the vulnerabilities that they respond to are, in part, shaped by the social, economic and political context and precarities experienced by people and communities. Digital exclusion exacerbates power imbalances that are often amplified through the design, implementation and availability of security technologies. Intersections between different types of forms of power and suppression can be uncritically and, at times, unconsciously embedded into technology design. Work from Cathy O’Neil [67] and Safiya Umoja Noble [65] are amongst the writings that have forcefully brought these intersections to the attention of wider audiences. The work of Slupska et al. have highlighted that intersections with power and suppression are also emerge in security technology design [91, 92]. Matthews et al.  [53] reveal how security technologies do not afford protection to those experiencing intimate partner violence. In their work Matthews et al. reveal how abusers might use technology such as GPS tracking, often marketed as offering protection to people, to exacerbate a power imbalance in a relationship and to increase one individual’s suppression of another.

### Enhancing Inclusivity

Participatory and critical approaches to security analysis and design are needed to highlight these biases and power inequities [27, 91] and the impact that these have on individuals and communities in marginalised and underserved communities accessing digital services by necessity. Of equal importance is using these critical and participatory methods to uncover the intersections between digital insecurity and other forms of social and economic security. Work at the intersections between digital security and other forms of insecurity has been particularly strong in studies that examine the security issues faced by refugees as well as studies that examine the security issues felt by prisoners [68] and homeless people [48, 90]. In their work with homeless people, Sleeper et al. looked at the intersections between financial insecurity, homelessness and digital privacy and security [90]. They identified the four factors that impacted the digital security and privacy of an individual experiencing homelessness and financial insecurity. These factors were: limited financial resources, limited reliable access to digital devices and the internet, the ongoing need to manage untrusted relationships and ongoing stress. In this work the intersections between digital insecurity and financial and social insecurities is particularly striking and underscores how deficits in social and economic capital and disabilities are often drivers of homelessness [87].

### Summary

In this section, we have reflected the breadth of issues that an inclusive security approach must address in the digital context. We have also shown how social, economic and political insecurities can both intersect with digital insecurities and exacerbate any ways in which a user might be disabled in using security and privacy technologies. The examples of contexts of technology use given in this section also reveal the extent to which security technologies need to be designed both for universal use and also for the security issues of particular circumstances if they are to be of benefit to all. It is this tension that inclusive security must navigate. One of the fundamental ways of addressing this tension to ensure that security and privacy technologies are designed in such a way that they are accessible and usable by users with a broad spectrum of abilities with differing levels of resource and a range of capabilities.

## Accessibility and Vulnerability

The W3C argues that an improvement in accessibility benefits all users, including those without disabilities [110]. Accessibility is a legal mandate [47]. The United Nations Convention on the Rights of Persons with Disabilities^1^, adopted in December 2006, is the first international legally binding instrument that sets minimum standards for the rights of people with disabilities. The UN has 193 member states, almost all countries on this planet, so this can be considered to be a global requirement. Even so, delivering accessibility is non-trivial.

2020 was declared the year of Digital Accessibility in the European Union (EU) with Anderson [6] reporting that the EU enacted a directive that makes accessibility compulsory for websites published by all public sector bodies and institutions that are governed by a public authority. Examples are public universities, local governments and any publicly-funded institution. There is much work still to be done to satisfy this directive [47]. However, as the number of court cases increase, it is likely that public institutions will be forced to take accessibility more seriously and find ways to design accessibility into their online-facing public services. The accessibility charity SCOPE [83] says: “*Removing these barriers creates equality and offers disabled people more independence, choice and control.*”

Persson et al. [70] point out that there is little or no consensus on a definition of accessibility. They report that even in ISO’s 18,000 standards, where the term accessibility occurs in over 400 documents, there is no attempt to define accessibility. Indeed, the definition provided by [31, p.2]: *“Easily used or accessed by people with disabilities: adapted for use by people with disabilities”*. is a case in point. While being concise, it leaves one with questions about what the word ‘disabilities’ encompasses. Persson et al. conclude their discussion by arguing that any discourse on accessibility should reflect the fact that the concept reflects “*flexible, ever-changing gaps between a person’s ability and a potential activity in a changing environment*” (p.523). The person could have permanent or temporary accessibility challenges, and the environment could prove more or less challenging with adaptations that are implemented over time. This formulation is flexible enough to accommodate this dynamism.

The International Classification of Functioning, Disability and Health explain that it is possible to distinguish a *social model of disability* rather that focusing primarily on a medical disability perspective. They explain that whereas a bodily disability can disrupt functioning, so can a mental disturbance, with no outer manifestation. Persson et al. [70] highlight the multidimensionality of the disability concept, and the fact that it manifests wherever there is a limitation to interaction with other people, the environment or artefacts in the environment. This social perspective to designing for accessiblity is also espoused by Gilbert [31]. Essentially, as argued by Shinohara et al. [88], designing accessibility from a social perspective humanises the people you are designing for.

We will thus rely on the ‘social model of disability’ defined by SCOPE [83], which explains that: “*disability is caused by the way society is organised, rather than by a person’s impairment or difference. Barriers can be physical, like buildings not having accessible toilets. Or they can be caused by people’s attitudes to difference, like assuming disabled people can’t do certain things. The social model helps us recognise barriers that make life harder for disabled people (both permanent and temporary disabilities)*”. This formulation highlights the fact that disability is not only physical, but also related to a range of barriers that prevent people from operating as fully fledged members of society.

### Vulnerabilities

When it comes to considering cyber security accessibility from a social perspective, we need to widen our focus beyond physical disabilities, which are the focus of most accessibility guidelines and laws. Cyber security is essentially risk management, actions to be taken to reduce vulnerabilities, i.e. the probability of falling victim to an attack via digital means. Hence, in discussing accessibility and inclusivity in the cyber security domain, we focus on *vulnerabilities*, which arise from a lack of access to resources or barriers to using technology in the way for which it is designed.

**Fig. 1:**
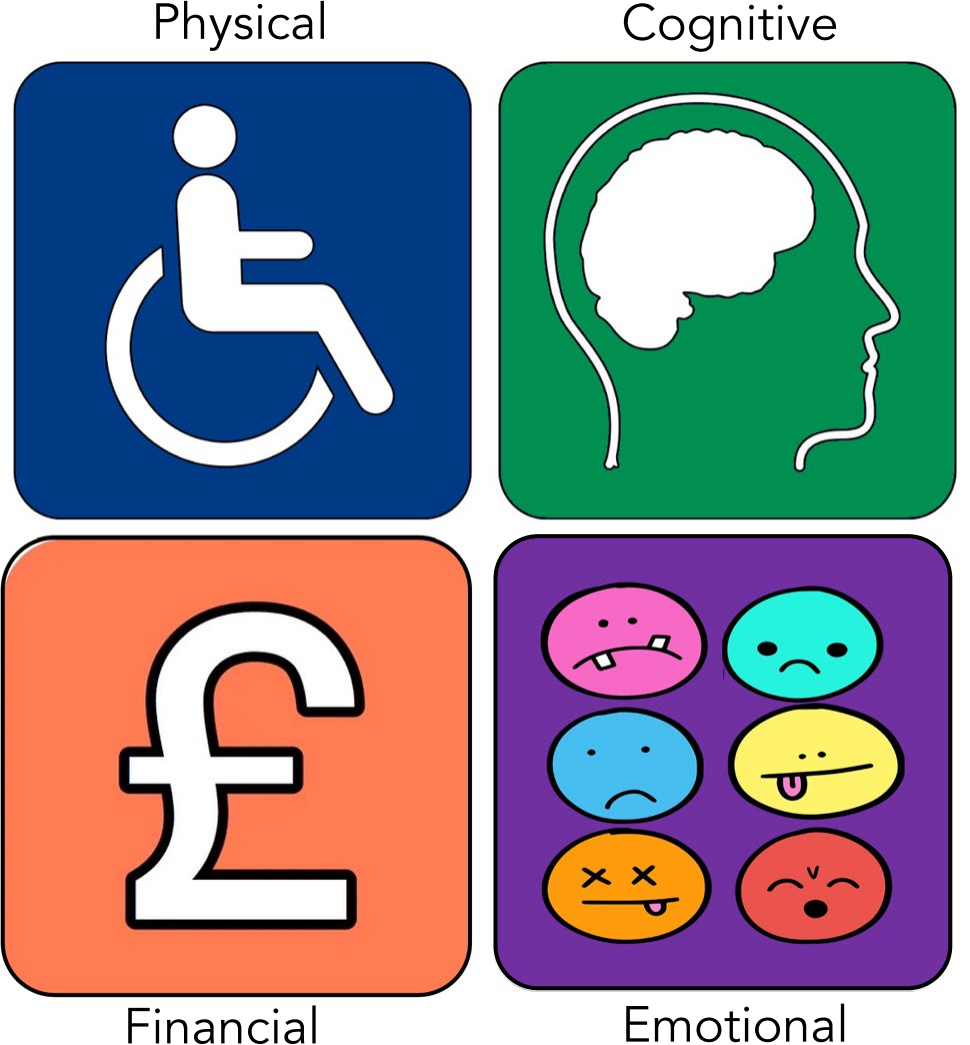
Vulnerability types considered

Numans et al. [66] offers a wider lens: that of *vulnerability*. They suggest three primary kinds of vulnerability: (1) mental (psychological), (2) physical and (3) financial. They also mention the feelings that co-exist with vulnerability, which arguably constitutes a fourth kind of vulnerability: emotional (see Fig. 1). The next section will briefly consider each of these in turn.

#### Cognitive

**Fig. 2:**
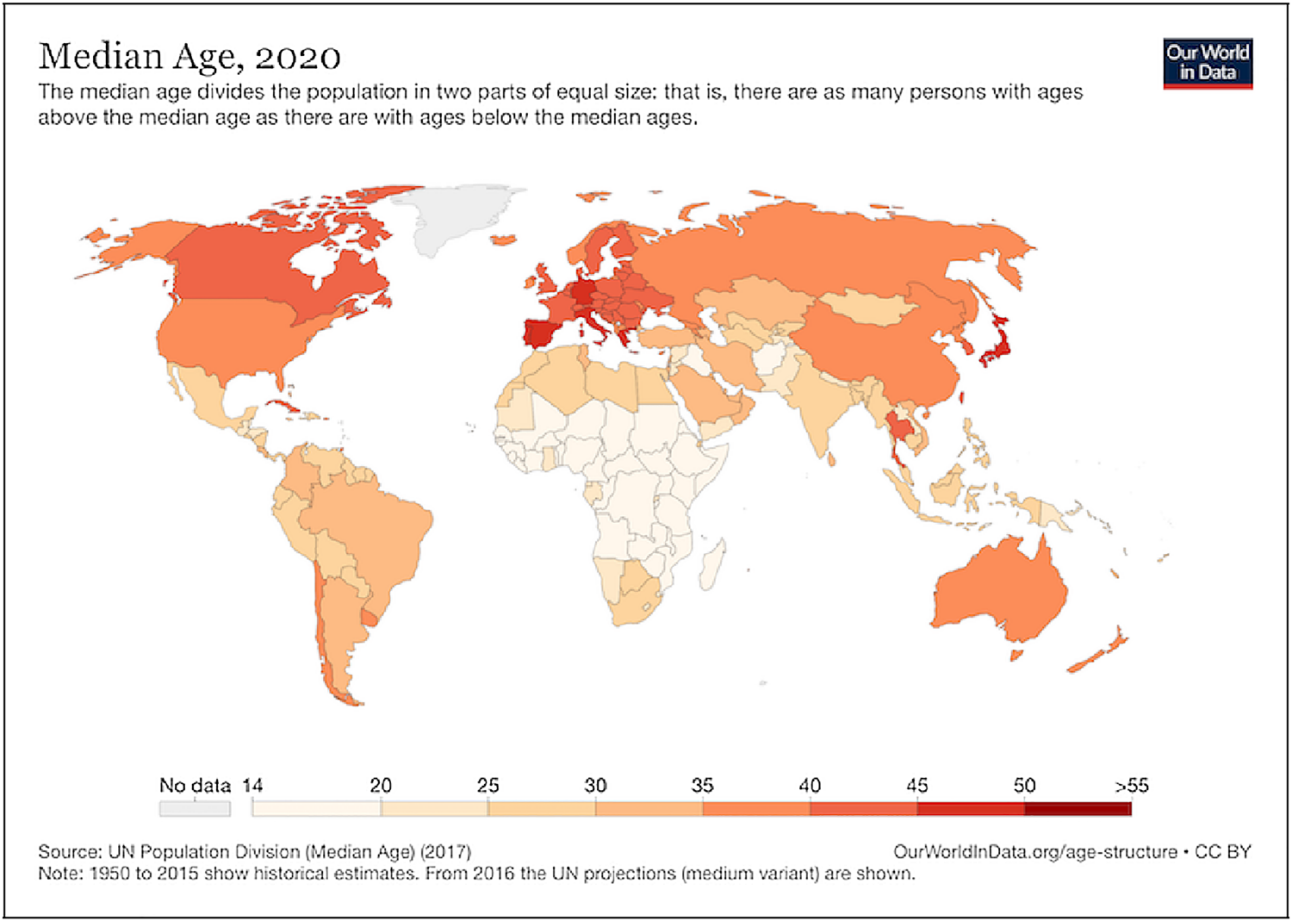
Median age of World population in 2020

A variety of cognitive disabilities are listed on the WebAIM website including: memory, problem-solving attention, reading, linguistic, and verbal & visual comprehension. The world’s population is ageing, as shown by Fig. 2. Many older adults experience a measure of cognitive decline [56]. For example, dyslexia impacts at least 10% of the population, and impacts cyber security behaviours [54, 76]. Users with other limitations, such as those with Down syndrome, need more time than other users to carry out tasks [52]. Other mental disabilities are likely also to constrain the types of data and technological practices that can be carried out [55, 58, 104].

#### Physical

“Physical Disability” includes people with visual & auditory impairments as well as motoric disabilities [6]. Anderson [6] reports that it is estimated that, in Europe, there are over 100 million people with disabilities of various kinds. We will now briefly consider the different kinds of disabilities.

**Vision and Auditory Disabilities:** Some users are completely blind, others have limited vision, and the WebAIM Website (Web Accessibility in Mind) website^2^ also lists colour blindness as a disability. If web designers use colour to highlight important messages, this is likely to be missed by colour blind users. Some people are born with poor or no vision, but many people develop vision and auditory issues as they age [101]. Worldwide increasing life spans suggest that the number of people without perfect vision and impaired hearing is steadily increasing.

The heavy dependence of modern day graphical interfaces on visual cues is problematic for the visually impaired [18, 109] and blind users face a large number of barriers to usage [50, 94]. Chiang et al. [18] cite Scott et al. [84], who carried out a study with people suffering from age-related macular degeneration. This ailment leads to visual impairment and severe vision loss. It impacts the centre of the retina, which is crucial in giving us the ability to read and parse text. Scott et al. report that the reduced visual acuity, contrast insensitivity, and decreased color vision impacted task accuracy and task completion speed. With particular application to authentication, Dosono et al. [24] review the difficulties visually impaired users face in this respect. Issues include locating the login pane on the web page, not being able to find relevant information related to password requirements and verifying that authentication has been successful. Moreover, password masking (displaying a $\bullet$ when a key is typed), interferes with the ability of users using assistive technologies, such as JAWS screen readers [24].

While Braille keyboards may help those who have been blind from a young age, Braille is not taught to those who lose their vision due to age-related decline or accidents during adulthood, so this is not necessarily an option for them. Moreover, with more people accessing the Internet from their Smartphones every year (see https://ourworldindata.org/age-structure), and thus interacting with security mechanisms via soft keyboards, poor vision can present insuperable barriers to usage, unless the mechanism designed with accessibility in mind. Some noteworthy solutions explicitly accommodate this demographic [24, 36, 39, 93, 117].

There is also evidence that deaf users and those with hearing impairments experience self-efficacy challenges when it comes to information security [62]. Murbach found that deaf users had poor security knowledge (confirming [46]), poor security behaviours and needed a support network to cope with information security. Fajardo et al. [29] presents a search engine that supports the use of sign language to carry out a search, a welcome movement in the right direction to make web searching more accessible to deaf users.

**Motoric Disabilities:** As people age, their dexterity decreases, especially after 65 [15]. Together with age-related vision loss, this is likely to impact their ability to engage with computer keyboards, both traditional and soft (on Smartphones). The WebAIM website lists a range of other motor disabilities, including multiple sclerosis and cerebral palsy. People with these disabilities are likely also to experience difficulties interacting with keyboards, computer mice and trackpads. Given that authentication is mostly achieved by requesting a person to type in a password, it is easy to see how people with motoric issues will struggle to do this correctly [7].

#### Financial

Numans et al. [66] mention the vulnerabilities caused by financial deficits. People might need to spend significant amounts of their funds due to some physical disability [61], with less money being left to spend on cyber security technologies such as virtual private networks (VPNs) or password managers. Finally, there is also the issue of those with financial deficits not having access to cyber security training, perhaps because they are not in employment. The knowledge gap between those who are in employment, and those who are not, is bound to widen inexorably as new exploits emerge.

Unemployed people, currently 4.7% of the UK population^3^, face particular challenges when it comes to cyber security. Seabright [86] explains that the unemployed inhabit ‘information islands’: there are no bridges to up-to-date information. This means that those who know a little inform others, and are not aware that they either misunderstand or are out of date. Society, Seabright says, does not construct bridges to these increasingly isolated societies. This is even more damaging in the cyber security context, a field that changes extremely quickly due to the continuous efforts of global cyber criminals coming up with new exploits.

Some people with severe disabilities are unable to work due to their health issues. Seabright [86] explains that it is often the case that healthy people make choices on behalf of these people. This increases the sense of isolation they experience. In the cyber security context, relying on others usually means giving the other person their access control credentials and having to trust in their integrity. Such trust is justified in the majority of cases, but not all [37, 103].

#### Emotional Vulnerabilities

Renaud et al. [78] carried out a study to uncover emotions related to cyber security and found that negative emotions are prevalent. Such emotions are bound to be unhelpful, and are not conducive in terms of encouraging people to take cyber security actions. The authors did not attempt to reveal the source of these negative emotions, but a number of causes could play a role.

In particular, we know that fear appeals are widely used to persuade people to implement cyber security measures [74]. Two aspects of these fear appeals make them less than efficacious. The *first* is that they fail to target the level of fear they trigger with any accuracy; they aim to trigger fear, and they do, but there is evidence that too little fear and too much fear can be counter productive [17, 95]. If too much fear is induced, recipients might engage in ‘fear control’ [19], i.e. avoiding the topic altogether.

The *second* aspect revealed by Renaud and Dupuis [74] is that none of the research studies into the use of cyber fear appeals ascertained that the recommended action they were trying to encourage was feasible to the recipient. This is particularly pertinent to the topic of this discussion. People may know what to do and know how to do it in the abstract, but still be unable to carry out the activity due to an emotional disorder.

Upsetting cyber experiences are also bound to leave long term impacts on the psyche, which will negatively impact future engagement with a range of data and technology protection practices [8, 72, 100].

Finally, very few cyber security professionals acknowledge the impact of a person’s existing (pre-training) cyber security practices, which undeniably exert pressure on them not to adopt new practices [3, 35, 77]. Any attempt to denigrate an existing practice will trigger a defensive response and will be likely to prevent adoption of new advised behaviours.

### Disability Standards and Cyber Security

The W3C’s Web Accessibility Initiative (WAI) has published a standard for web accessibility called the Web Content Accessibility Guidelines (WCAG) [111]. WCAG 2.1 (published in June 2018) did not really address cyber security accessibility. Only one instance can be found which refers to the need to provide users with enough time to read and use content, and the ability to pick up an activity they were previously engaged in after re-authenticating an expired session (success criterion 2.2.5).

WCAG 2.2 introduces a new success criterion called ‘Accessible Authentication’ (3.3.7). This specifies that “*for each step in an authentication process that relies on a cognitive function test, at least one other method is available that does not rely on a cognitive function test*” [112].

WCAG [115] explains that a cognitive function test is: “*a task that requires the user to remember, manipulate, or transcribe information*”. Remembering a username and password (or any other secret used by a knowledge-based authentication mechanism) is such a test. The alternative authentication method must *not* rely on human cognition. It might be a password manager automatically filling in credentials [40] or a biometric [82], for example. Sometimes, authentication requires multiple steps. In this case, all steps should comply with this success criterion.

## Barriers to Accessible Cyber Guidance

In this section, we examine the barriers and challenges to developing accessible cyber guidance. In conducting this analysis we are highlighting the fundamental challenges to ensuring that universal cyber security guidance is accessible and usable by all. We will ground these in the UK’s National Cyber Security Centre’s Cyber Aware advice.^4^ This list is admittedly limited, and does not claim to cover the full extent of cyber security actions that individuals should engage in. Cain [14], for example, provides a much more extensive list. However, for the purposes of this discussion, the NCSC list does provide a set of recommended actions that we can evaluate to demonstrate the kinds of accessibility issues people can face in carrying these out.

The NCSC provide six items of cyber hygiene advice:

1. **Use a strong and separate password for your email.** Being able to do this assumes that the person has the mental ability to create strong and unique passwords, something which is not possible for many of those with cognitive disabilities (See Sect. 3.1.1) [34]. Entering a password might be challenging for those with dexterity issues such as arthritis, people with temporary paralysis such as Guillain–Barré syndrome, or people who have lost fingers. Entering any password using a soft keyboard requires good vision and slim enough fingers not to press multiple or incorrect keys on the small keyboard.
2. **Create strong passwords using three random words.** This assumes a basic level of literacy, which some may not possess, especially when they are pre-literate, or have not had the benefit of an education. Moreover, many older people, even if well educated, lose the ability to maintain attention for long enough to enter a password without feedback, even if they are able to memorise it. Lobo et al. [51] found that visually impaired users were more likely to use predictable passwords, to disable PIN access to their Smartphones and were unable to get past CAPTCHAs to access their accounts (confirming [89]). Users with some kinds of cognitive disabilities need more time to create passwords [52]. Ma et al. [52] advocate giving these users a choice of their preferred authentication mechanism to enhance accessibility. There has been a move to the use of biometrics by more expensive devices, such as iPhones. Some biometrics are not ubiquitous e.g. fingerprints, which can degrade with age or due to medical treatments [32]. Other biometrics are more ubiquitous, but often suffer from unacceptable bias issues [116].
3. **Save your passwords in your browser.** This advice relies on an assumption of ‘one user: one device’. For many of those on low incomes, sharing of devices is likely. This means that this particular practice is probably contra-indicated in this context.
4. **Turn on two-factor authentication (2FA).** Two factor authentication often requires a separate device. These operate in one of two ways: (1) *receive a one-time code*. This requires ownership of a mobile phone, which seems reasonable in many developed countries. Some two factor authentication mechanisms make use of physical tokens, which have to be purchased, so those with financial limitations might not be able to afford one. It also requires the person to be able to re-enter the delivered code into the user interface, an assumption that does not hold for the entire population. This option requires the user to read and correctly transfer the number to the device they are logging in on. These codes expire within seconds/minutes so even a slight delay caused by age-related slowness might invalidate them, which will eventually lock the user out of their account [76]. (2) *approval of a login attempt via a 2FA app*. This option requires a Smart phone, which is out of reach for those on low incomes. Finding the right button to press might be difficult for those with visual difficulties. Some of these apps require the user to respond within 30 s, which will catch out many disabled users. To accommodate age-related and other cognitive disabilities [52], timeouts on two factor authentication mechanisms ought to be configurable.
5. **Update your devices.** Updates can only be carried out if the device itself is modern enough and has enough hard drive space to sustain it. Moreover, previous negative experiences will deter people from installing updates [107]. Those using old devices might not be able to follow this advice, and their devices will thus be vulnerable to exploitation. For example, consider the following fictitious but realistic scenario. A Windows machine has software installed that allows it to operate an MRI machine. This particular software developer is no longer in business and not issuing updates. It still does its job well despite this. Updating the operating system breaks this software. The hospital has the choice of: (1) installing the update and not being able to use the MRI machine any more, or (2) not installing the update. In this scenario, one can see that the obvious choice is the latter.
6. **Back up your data.** This is a great piece of advice, but has financial implications. A number of cloud options are available, but these often require a level of expertise that might not be possessed by users. Moreover, there are space limitations with more space needing to be paid for monthly, which might be out of the reach of those with financial limitations. A good way of backing up is to use an external hard drive but this is an extra expense, and people then have to have a secure space at their disposal to secure these in their homes, which might not be feasible given their living environment.

In this review, we have not mentioned emotional aspects because they might easily apply across the board. This might be because fear is deliberately used to encourage precautionary actions [11], because an action has previously led to negative outcomes and emotions [107] or because they lack self confidence, which means that many security-related activities trigger negative emotions [114] (Table 1).

**Table 1:** Accessibility challenges of NCSC advised actions

Advice	Physical	Cognitive	Financial	Emotional
Use a strong and separate password for your email	$\bullet$	$\bullet$		$\bullet$
Create strong passwords using three random words	$\bullet$	$\bullet$		
Save your passwords in your browser			$\bullet$	
Turn on two-factor authentication (2FA)	$\bullet$	$\bullet$	$\bullet$	$\bullet$
Update your devices			$\bullet$	$\bullet$
Back up your data		$\bullet$	$\bullet$	$\bullet$

As we can see, there are accessibility issues with all of these items. We do not aim to criticise the NCSC—this advice is sound and valuable. We merely use this list to demonstrate the difficulties that can be experienced by those with particular vulnerabilities in the cyber security domain.

## Signposting the Way Forward

**Fig. 3:**
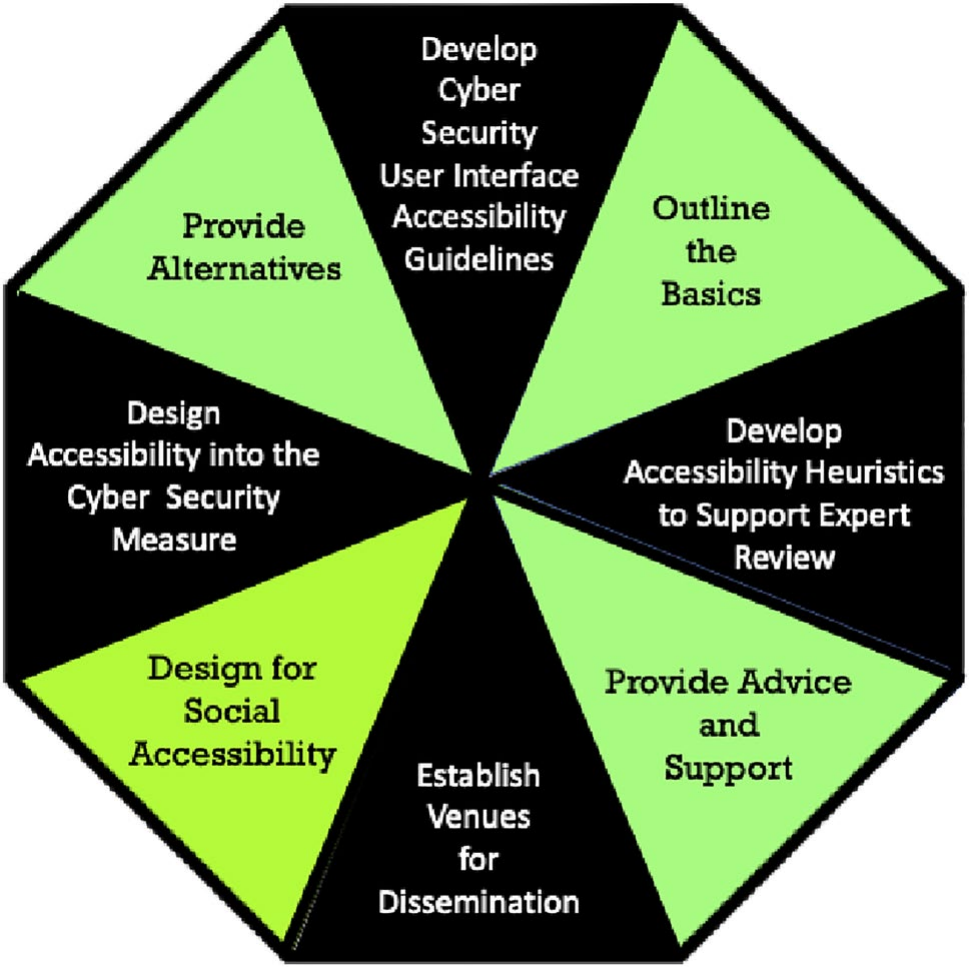
Signposts towards accessible and inclusive cyber security

Governments are increasingly offering services online, so that their citizens, both abled and disabled, have no choice but to go online as well. This means that they will also interact with cyber security mechanisms and measures during their everyday lives [4]. Hence, everyone working in cyber security has to consider accessibility when designing and deploying security measures. Those designing these measures have to ensure that they do indeed provide the required level of security, but also that they maximise both usability and accessibility. Figure 3 provides an overview of future avenues of research that will be suggested in this section.

We do not pretend to have solutions. We merely point to the pressing need for designers to produce accessible and inclusive security solutions. This will require concerted efforts from determined and talented researchers. It is fortunate that the usable security and inclusive security research fields has many of these.

In this section, we suggest some directions for future research, with no claims to exhaustiveness. We hope that other researchers will take up the accessibility challenge and carry out research to improve accessibility for all users. Duarte [26] highlight a number of innovations with new technologies which can make a difference in this space. First steps towards accessible and inclusive cyber security (first enumerated in [73]) are:

1. **Outline the basics:** One of the standard accessibility guidelines is to ensure that alt-text is provided for all visuals. In the cyber security domain, for example, if a visual nudge is provide, such as a password strength meter, those with poor vision will not be able to see what this is trying to communicate. An alternative to a visual communication measure should always be provided to ensure accessibility. However, this is too specific to cover the entire cyber security domain. Gilbert [31] provides a meta-level perspective: (1) Who is using your product (and what user vulnerabilities do you need to accommodate)? (2) What are they doing (and what are they able to do)? (3) Where are they doing it (how will the context influence their ability to complete the task)? (4) When are they doing it? (5) Why are they doing it (is it optional or compulsory)? (6) How are they doing it (what devices are they using)? The cross-cutting theme here is that security considerations have to be maintained, so we should add: (7) what are the security requirements of the user’s actions?
2. **Design for social accessibility:** Shinohara et al. [88] propose three design tenets in the accessibility space, which apply equally here: (1) incorporate target users, both with and without disabilities during the design process ([38, 60, 81]), (2) address functional and social factors simultaneously [118] and (3) include tools to bring social factors in accessible design to the forefront during the design process [9, 13].
3. **Provide alternatives:** The WCAG guideline already mandates an alternative to authentication. This principle ought to be applied to other measures too. So, for example, the visual display of a password strength meter should offer an audible or haptic feedback measure for users with poor vision. CAPTCHAs often provide an audible alternative but for ageing users with both vision and hearing impairments this is probably not going to be sufficient, especially since both of these add ‘noise’ to prevent automated solving. Such noise makes it very difficult for those with imperfect vision or hearing to decipher the actual signal. Finding an alternative would be a good avenue for future research. The use of biometrics, in particular, should be investigated for more widespread use. Some consumers already actively use face and other biometrics to authenticate to their phones. With increasingly powerful built-in cameras on a range of devices, it seems as if biometrics’ time has come, in terms of providing a usable and accessible alternative. Some initial moves in this direction are encouraging [33, 45, 99].
4. **Design accessibility into the cyber security measure**: what we have learnt is that accessibility, similar to security and usability, cannot be bolted on at the end of the design and testing process. It has to be a consideration all the way through the requirements gathering, design, development and testing parts of the life cycle. Hence, cyber-security related software design guidelines are needed. Testing should be carried out with disabled as well as able users. Kerkmann and Lewandowski [44] provide practical guidelines for researchers who want to conduct an accessibility study. Theirs is specifically aimed at web accessibility but would provide a good starting point for developing similar guidelines for testing the accessibility of cyber security mechanisms.
5. **Develop cyber security user interface accessibility guidelines**: McCarthy et al. [54] point to the lack of guidelines for usability testing to accommodate the needs of dyslexics. There is a need for guidelines to cover security interface design and testing. Being able to quantify the accessibility of a particular interface, as suggested by Vigo et al. [108], will support comparisons, which would be helpful. Levin and Hepler’s [49] have developed design guidelines for interfaces that specifically accommodate the needs of those with low digital literacy. It is likely that these could be extended for those with low cyber literacy as well, and this would be a fruitful avenue for future research. Some authors have already started experimenting with such three-way evaluations, e.g. [22, 28, 42]. We can start with the WCAG accessibility guidelines, and then extend them to encapsulate the cyber security domain. For example, there is now a requirement for captioning on all multimedia, and a number of successful court cases have ensured that companies realise this [23]. If an organisation chooses to raise Cyber Security awareness using an online course, which includes videos, these *must* be captioned. Moreover, few of these support questions from viewers, which is an omission that should be addressed.
6. **Develop accessibility heuristics to support expert review:** The usability field has developed a range of heuristic guidelines to support expert review of interfaces [59, 64]. The idea would be to develop a similar range of heuristics for accessibility assessment of cyber security measures. Napoli et al. [63] have proposed an initial set of heuristics for this purpose, and it would be good to see others building on these. This will help businesses to redesign their cyber security measures that users have to interact with to ensure accessibility too [5].
7. **Evaluate technologies at the intersections**: As we highlighted in Sect. 2, marginalised and underserved groups often operate at the intersections of a range of insecurities. It is therefore important that technology testing and evaluation takes these intersections into account to ensure that the proposed technology design does not exacerbate existing vulnerabilities, and can be used in contexts that are shaped by a range of precarities. Testing and evaluation approaches that are sensitive to the intersections between insecurities also contribute to the wider discussion about the deployment programmes and processes in which to embed digital technology and service roll out.
8. **Establish venues for dissemination**: the establishment of conferences such as SOUPS, STAST and EuroUSEC have played a role in encouraging research in the usable security domain. We need similar conferences for accessible security too, or at least dedicated streams in other human-related conferences, such as the huge and successful CHI conference.
9. **Provide advice** *and* **support**: one of the stakeholders in this domain is government, especially those governments who cyber responsibilise their citizens [75], i.e. issuing a great deal of advice and leaving people to get on with it, without support. Given that vulnerable users may struggle even more than others to act on any cyber security advice that is issued, there is a clear need for more support to be provided to them. The way this ought to be provided is yet another rich avenue for future research.

## Conclusion

Cyber security is a relatively new field, and efforts to improve its usability are barely two decades old. As the field of human-centred security matures, it is appropriate for us also to consider accommodating the needs of *all* digital technology users: to make accessibility one of our primary aims as we design security systems. Our efforts to improve accessibility are bound also to make cyber security more manageable for the rest of the population, in addition to enhancing access for those with vulnerabilities. With this paper, we hope to raise awareness of the need for more research in this area. We trust that human-centred security researchers will bear accessibility in mind in their future research endeavours.

## Footnotes

1 https://ec.europa.eu/social/main.jsp?catId=1138 &langId=en.

2 https://webaim.org/articles/motor/motordisabilities.

3 https://www.ons.gov.uk/employmentandlabourmarket/peoplenotinwork/unemployment/timeseries/mgsx/lms.

4 https://www.ncsc.gov.uk/cyberaware/home.

## Declarations

### Human and animal participants

We did not involve any human participants in this study.
